# Newborn Critical Congenital Heart Disease Screening Using Pulse Oximetry: Value and Unique Challenges in Developing Regions

**DOI:** 10.3390/ijns6030074

**Published:** 2020-09-15

**Authors:** Lisa A. Hom, Gerard R. Martin

**Affiliations:** Children’s National Heart Institute, Children’s National Hospital, Washington, DC 20010-2970, USA

**Keywords:** CCHD screening in developing countries, newborn screening pulse oximetry, critical congenital heart disease screening

## Abstract

Newborn screening for critical congenital heart disease (CCHD) is recommended for implementation in many developed countries as the standard of care. Efforts to implement this point of care screen in developing regions face unique barriers, and present important opportunities. The First Pan-African Newborn Screening Conference, held in Rabat in June 2019, incorporated a workshop dedicated specifically to identifying and discussing CCHD screening issues in the Middle East Northern Africa (MENA) region. The issues explored may be beneficial as part of the greater discussion of CCHD screening’s growing importance in developing regions around the world. Screening experts presented education and lessons learned from previous CCHD implementations, including a hands-on technical demonstration of CCHD screening. Children’s HeartLink, The Newborn Foundation, and Children’s National Hospital each presented on their experiences working with teams and pilot projects from around the world. Experience in implementation from Children’s Hospital Marrakesh was presented and highlighted some of the unique findings, challenges, and experiences of screening in Morocco. As developing regions investigate the implementation of CCHD screening using pulse oximetry either as part of research studies, pilots, regional studies, or as part of a nationally supported program, data to inform policymakers on the benefits of screening and specific needs for infrastructure development and resources are essential. This special issue contains initial lessons learned on newborn CCHD screening from a select number of developing countries, including Saudi Arabia and Morocco and regions such as Latin America.

Newborn critical congenital heart disease (CCHD) screening using pulse oximetry is well established and implemented in many developed countries either through mandates (with an increasing number of countries having national recommendations) or as the standard of care [1]. The identification of infants with CCHD prior to circulatory collapse has been shown to improve outcomes [2]. CCHD screening has been shown to identify many of those infants not found by prenatal testing or newborn examination who are at greatest risk for circulatory collapse when not identified during the transitional circulation.

Critical congenital heart disease (CCHD) occurs in roughly 2–3 out of every 1000 live births [3]. Higher birth rates in low- and middle-income countries increase the burden of disease in these parts of the world. The Lancet recently published an analysis investigating the global burden of congenital heart disease (CHD), concluding that CHD is responsible for over 260,000 deaths annually worldwide [4]. The majority of those deaths occur in infants less than 1 year of age and in countries associated with low or middle Socio-Demographic Index (SDI) quintiles [4,5]. Outcomes for CHD improve correspondingly in regions with higher SDI quintiles [4]. Roughly 90% of infants born with CHD are born in regions with limited or no access to care and in areas where mortality remains significantly higher than the 90–95% that survive into adulthood in developed countries [4]. This disparity in outcomes was dramatically captured in a recent analysis examining the global burden of disease over the past 15 years. In a list of the top ten causes of death in infants 1 month to 1 year in age in middle socio-demographic index nations, deaths attributed to CHD rose from being the fourth leading cause to the second leading cause just after lower respiratory infections [4,6]. Reasons for this are multi-factorial and include improved global health measures in the areas of vaccination rates, access to safe water, improved maternal education, and a decrease in poverty associated diseases such as diarrheal diseases [7]. As mortality from communicable diseases has improved, non-communicable diseases, such as CCHD are becoming a more significant contributor to infant mortality.

Access to medical care is more challenging, with many infants born with CHD going largely undiagnosed, and those with critical forms rarely receiving the medical interventions that can save or dramatically improve their lives [8]. In many low and middle, SDI countries access to any form of surgery is challenging and even more so access to pediatric cardiac surgery [7]. Other challenges facing low resource settings include a high proportion of infants who are born at home or in very resource-limited government hospitals, limitations in equipment availability, infrastructure, and trained medical personnel, as well as high volumes of deliveries [9]. Prenatal screening and newborn screening programs are often not well developed, with limited access to echocardiography and limited treatment options [9]. There are also socio-cultural factors that may impact the recognition of an appropriate diagnosis as well as referral [7,9]. Once a diagnosis is made, stabilization, transport, and treatment are impacted by poorly developed newborn transport systems [8,9]. The cost can be prohibitive, and pediatric heart programs that perform cardiac catheterization, or cardiovascular surgery may be geographically spread out and limited in number [7,9]. Kumar describes many of these health system barriers to delivering cardiac infant care in low resources settings, many of which remain largely unchanged since his 2016 manuscript was published [9].

Despite the limitations in access to care for infants with CCHD in much of the developing world, there are potential benefits to be gained by screening for CCHD using pulse oximetry. In June 2019, the Moroccan Ministry of Health, in conjunction with the Moroccan Society for Newborn Screening and Handicap Prevention, the International Society for Neonatal Screening (ISNS), the Pasteur Institute of Morocco, the National Institute of Health, the American College of Medical Genetics and Genomics, the US National Library of Medicine, Children’s National Hospital, the Newborn Foundation, the Association of Public Health Laboratories (AHPL) and other important organizations hosted the first Pan-African Newborn Screening Conference. During the meeting, a half-day workshop was dedicated to exploring and discussing CCHD screening, with a focus on issues facing Africa and the Middle East. The set of manuscripts contained in this special issue capture the content of some of the presentations from this workshop, as well as expanded findings from pilots and implementation projects on which the faculty of the workshop, Children’s HeartLink, The Newborn Foundation, and Children’s National Hospital, have collaborated in both the MENA region as well as globally.

Newborn screening initiatives, such as screening for CCHD using pulse oximetry, become increasing public health priorities in low and middle SDI countries as infant mortality rates (IMR) decreases [10]. The overall goal of the conference was to focus on topics specific to Africa and the MENA region where sustainable implementation is a difficult, but worthwhile, goal as congenital disorders are a growing concern in infant and newborn morbidity. This is particularly true as 40 of the 50 countries with the highest fertility rates are in Africa [10]. Additionally, the MENA region is associated with large family sizes and high levels of consanguinity [10,11]. Factors, including providing participants a venue to discuss, learn from international experts, and discover opportunities (based on recent advances in research and technology), were interwoven with providing a platform in which to discuss common challenges and share updates on implementation and successful intervention programs. Identification of enabling factors leading to successful and sustainable newborn screening programs included the need for governmental prioritization, sustainable funding, public acceptance, medical and health professional, as well as stakeholder coordination and promotion (Conference website: http://medicalintelligence.org/nbsm2019/) [10].

The CCHD screening workshop included a brief introduction to the goals of CCHD screening, as well as a history of implementation, the targets of screening, and the sensitivity of the screen. This basic background was followed by a pediatric cardiac nurse and a parent advocate presenting on the ‘Nuts and Bolts of Implementation’ and a how-to demonstration of how to screen, as well as education on the algorithm for interpreting results and required education and frequently asked questions (FAQs) for both healthcare staff and families of infants. A pediatric cardiologist provided important considerations on how to interpret screening results, as well as guidance on how to manage a positive screen. A focused presentation on building capacity in Africa and the development of infrastructure around pediatric cardiac health care capacity and care delivery was followed by an implementation update from a physician working at Marrakesh Children’s Hospital (Mohammed VI University Mother and Child Hospital). This presentation explored the preliminary outcomes, challenges, and implications for the region based on experiences in Morocco. A discussion panel and active participant sharing rounded out the half-day workshop dedicated to CCHD screening.

Several years ago, the *International Journal of Newborn Screening* released a special issue on CCHD screening, which provided manuscripts covering some of the broad aspects of CCHD screening using pulse oximetry. It included discussions on test accuracy in a variety of settings, including home births, at altitude, and using various protocols (foot only versus both pre- and post-ductal) [1]. Furthermore, it provided an overview of the benefits and challenges associated with CCHD screening and focused largely on settings within developed countries, which is where pulse oximetry screening was initially implemented at the population level and showed positive outcomes.

The focus of this special issue is the role and growing importance of CCHD screening using pulse oximetry in developing countries. It includes a manuscript from Saudi Arabia describing how a national program can be implemented incorporating the most up to date technology in data collection and based on current best practices with careful planning, structured preparation, and perhaps most critically—government support [12].

Although not presented at the CCHD workshop in 2019, also included is a manuscript from the Ibero American Society of Neonatology (SIBEN) [13]. It provides details on the national and regional collaboration of 13 countries in Latin America, which allowed them to move from a regional consensus statement to varying stages of implementation, including the training of nurses and physician staff. Early reports indicate that the impact in Latin America, while challenging to achieve, will result in significant improvements for infants born with CHD and CCHD, as well as those infants detected who have respiratory and hypoxic conditions [13].

Another unique manuscript included is the first CCHD screening feasibility pilot study conducted in Morocco, a country whose challenges include significant neonatal mortality (total overall neonatal mortality in the Marrakesh region is approximately 16 infant deaths per 1000 live births) [11], limited access to cardiovascular surgery, and ongoing efforts to develop pediatric cardiac intensive care expertise [11]. Telemedicine echocardiography consultations helped address the challenging need for access to specialists not available locally. A stated goal of the pilot was to improve the early detection of CCHD to enable better management. It is significant to note that similar to what Singh and colleagues [14] found in their retrospective review of CCHD pulse oximetry screening in the United Kingdom, where 79% of screening test positives identified significant clinical conditions, in the Morocco pilot, 86.7% (13/15 positive test results) of test positives identified important pathologies including five CCHD, five CHD, one PPHN, and two cases of neonatal sepsis [11].

Considerable expertise with capacity building for CHD has been gained over many years with Children’s HeartLink (established in 1969), The Newborn Coalition (initiated in 2009), and the National Health Mission of India (launched 2005). They report 66 countries with efforts currently underway in the form of pilots or partial to full implementation and describe five important considerations for those countries who wish to consider newborn CCHD screening in the future [15]. In the article, they argue newborn screening is a crucial early step in ensuring lifelong care for patients born with CHD. They emphasize the need to consider both local challenges and the local existing healthcare context [15].

As developing countries investigate the implementation of CCHD screening using pulse oximetry (as part of research studies, pilots, regional studies, or as part of nationally supported programs), capturing and sharing valuable lessons learned and outcome data are essential. This data and information will help to inform policymakers on the benefits and utility of screening, the specific needs for infrastructure development, and how to best utilize existing resources. An implementation map of the world illustrates how CCHD screening is spreading by grouping countries into several general categories. This includes countries that: recommend screening (either by medical societies or by government policy) (purple); have ongoing multi-center studies and/or pilot programs (orange); are interested in screening; are in the early stages of organization (blue); or where screening activity is unknown and likely not yet implemented (grey). This map has been used for the past eight years to help track global progress (Figure 1). 

Infants screened for CCHD using pulse oximetry may benefit greatly—not only through the identification of CCHD, but also from the detection of important secondary targets, such as respiratory and infectious diseases prevalent in neonates. Collaboration and data sharing should continue to be encouraged, since much of the CCHD global population will continue to reside in countries with low and middle SDI quintiles.

## Figures and Tables

**Figure 1:**
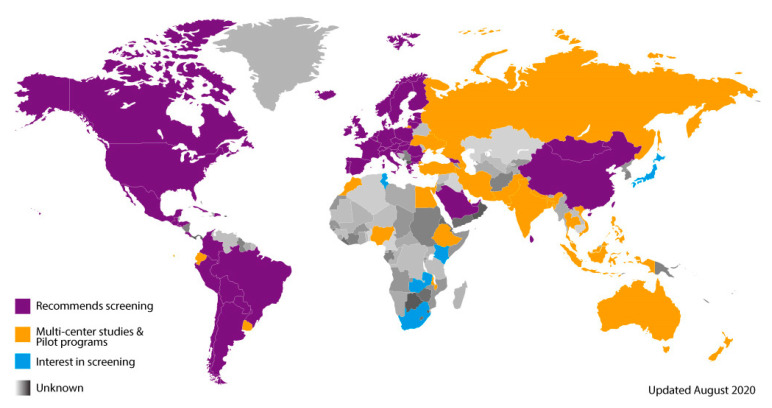
Critical congenital heart disease screening global implementation map.
